# Cost and efficiency of public sector sexually transmitted infection clinics in Andhra Pradesh, India

**DOI:** 10.1186/1472-6963-5-69

**Published:** 2005-11-05

**Authors:** Lalit Dandona, Pratap Sisodia, TLN Prasad, Elliot Marseille, M Chalapathi Rao, A Anod Kumar, SG Prem Kumar, YK Ramesh, Mead Over, M Someshwar, James G Kahn

**Affiliations:** 1 Health Studies Area, Centre for Human Development, Administrative Staff College of India, Hyderabad, India; 2 Andhra Pradesh State AIDS Control Society, Hyderabad, India; 3 Institute for Health Policy Studies and AIDS Research Institute, University of California, San Francisco, USA; 4 Development Research Group, World Bank, Washington DC, USA

## Abstract

**Background:**

Control of sexually transmitted infections (STIs) is an important part of the effort to reduce the risk of HIV/AIDS. STI clinics in the government hospitals in India provide services predominantly to the poor. Data on the cost and efficiency of providing STI services in India are not available to help guide efficient use of public resources for these services.

**Methods:**

Standardised methods were used to obtain detailed cost and output data for the 2003–2004 fiscal year from written records and interviews in 14 government STI clinics in the Indian state of Andhra Pradesh. The economic cost per patient receiving STI treatment was calculated, and the variations of total and unit costs across the STI clinics analysed. Multivariate regression technique was used to estimate incremental unit costs. The optimal number of STIs that could be handled by the clinics was estimated.

**Results:**

18807 STIs were diagnosed and treated at the 14 STI clinics in fiscal year 2003–2004 (range 323–2784, median 1199). The economic cost of treating each STI varied 5-fold from Indian Rupees (INR) 225.5 (US$ 4.91) to INR 1201.5 (US$ 26.15) between 13 clinics, with one other clinic having a very high cost of INR 2478.5 (US$ 53.94). The average cost per STI treated for all 14 clinics combined was INR 729.5 (US$ 15.88). Personnel salaries made up 76.2% of the total cost. The number of STIs treated per doctor full-time equivalent and cost-efficiency for each STI treated had a significant direct non-linear relation (p < 0.001, R^2 ^= 0.81; power function). With a multiple regression model, apart from the fixed costs, the incremental cost for each STI detected and cost of treatment was INR 55.57 (US$ 1.21) and for each follow-up visit was INR 3.75 (US$ 0.08). Based on estimates of optimal STI cases that could be handled without compromising quality by each doctor full-time equivalent available, it was projected that at 8 of the 14 clinics substantially more STI cases could be handled, which could increase the total STI cases treated at the 14 clinics combined by 38% at an additional cost of only 3.5% for service provision.

**Conclusion:**

There is un-utilised capacity in the public sector STI clinics in this Indian state. Efforts to facilitate utilisation of this capacity would be useful, as this would enable more poor patients with STIs to be served at minimal additional cost, and would also reduce the cost per STI treated leading to more efficient use of public resources.

## Background

India has one of the highest number of persons living with HIV in the world [1,2]. The presence of sexually transmitted infections other than HIV (referred to as STIs in this paper) increases substantially the risk of acquiring/transmitting HIV, and therefore, the control of STIs can be an important part of the effort to control emerging HIV epidemics [3]. The state of Andhra Pradesh with 80 million population has one of the highest estimated burden of HIV among the Indian states based on antenatal sentinel surveillance, and also one of the highest HIV prevalence (16–30%) among public sector STI clinic attendees included in the sentinel surveillance during 1998–2004 [4].

STI clinics in the public sector hospitals run by the government provide care predominantly to the poor segment of society in India, and therefore, their role in controlling STIs and HIV is particularly important. Recognising the important role that the public sector STI clinics could play in HIV control, the number of formally designated STI clinics in the government hospitals in Andhra Pradesh was increased from 28 to 85 in the year 2002 (oral communication, Andhra Pradesh State AIDS Control Society, Hyderabad, India, 14 December 2004).

With HIV/AIDS control having become a major public health issue in India [5], the funding available for this has been increasing [6,7]. However, assessment of the cost and efficiency of various strategies to control HIV in India is not readily available [8]. This information is needed for efficient utilisation of resources available for HIV control. As part of a study to assess the cost and efficiency of various HIV prevention strategies in Andhra Pradesh, we assessed the cost and efficiency of STI clinics in the public sector.

## Methods

This study was part of a larger multi-country effort to study cost and efficiency of HIV prevention in India, Mexico, Russia, South Africa and Uganda by the Prevent AIDS Network for Cost-Effectiveness Analysis (PANCEA) [9]. Details of the methods for the overall multi-country study are described elsewhere [10]. Description of the methods relevant for this paper follows. This study was approved by the Ethics Committee of the Administrative Staff College of India, Hyderabad, India and the Committee on Human Research of the University of California, San Francisco, USA.

### Selection of STI clinics

We included STI clinics in the government-run public sector hospitals for this study. These clinics provide services predominantly to the poor at no or minimal fee, and many persons receiving services here have relatively advanced STIs. At the time of starting data collection for this study in mid-2004, 85 public sector clinics were functioning. Of these, 28 STI clinics had been functioning since the 1960's in the state capital and the district headquarters as part of the medical college hospitals and other major public hospitals of Andhra Pradesh. In addition, 57 STI clinics were formally designated in mid-2002 in public hospitals, in district headquarter hospitals and in area hospitals located in smaller jurisdictions. Generally, the STI care in medical college hospitals and district headquarter hospitals is provided in distinct separate clinics, whereas in area hospitals it is provided as part of the general outpatient.

Andhra Pradesh has three geographic regions: the northern Telangana region has the state capital and nine other districts, the eastern Coastal region has nine districts, and the southern Rayalseema region has four districts with a population of nearly half that in the other two regions. In order to obtain a broad sample of STI clinics in public hospitals, we used the three geographic regions of Andhra Pradesh and the old-new clinics as the two strata for sampling. Fifteen STI clinics were randomly sampled to obtain six clinics each in the Telangana and Coastal regions and three clinics in the Rayalseema region, which included three each of old and new clinics in Telangana and Coastal regions and two old and one new clinics in Rayalseema region.

### Data collection procedures

The initial versions of the data collection instruments from the global PANCEA study were reviewed and adapted to suit the context of Andhra Pradesh. The data collection team, consisting of six researchers with background in economics or finance, was involved with the adaptation of the instruments and received extensive training to ensure a standardised approach to data collection. A pilot study was done to make final refinements in the data collection format and approach.

Data were collected for the April 2003 – March 2004 fiscal year at the 15 sampled STI clinics during June – August 2004. Data collection included a history of the evolution of the STI clinic, detailed cost and output data by month, and patient exit interviews. Formal informed consent to collect data was obtained from the senior-most person responsible for each STI clinic, generally the superintendent of the hospital in which the clinic was located. The STI clinic medical officer(s), medico-social worker, counsellor, and laboratory technician were interviewed and available written records reviewed to obtain data. Each visit started with an interview containing structured open-ended questions on the history of the STI clinic, and operational or community factors that may have affected the demand for or supply of services. Data collection then proceeded to cost and output data, in parallel with exit interviews of patients. Data collection at an STI clinic by three investigators lasted one week. Data were recorded in the field on laptop computers in MS Excel and MS Word files, which after review were entered into an MS Access database.

### Cost data

The cost of the STI clinic was divided into five categories: salaries, recurrent goods, capital goods, recurrent services, and rentals. These cost data were collected for each month, as far as possible. Economic cost was computed, i.e. the true resource cost incurred rather than just the financial cost. The inpatient cost was also included for the inpatient facilities that were being utilised for some of the STI clinic patients. Similar costing methods were used across the clinics for the five cost categories.

Salary cost was computed for all personnel contributing to the work of the outpatient and inpatient services of the STI clinic, which included the medical officer(s), medico-social worker/counsellor, nurse(s), laboratory technician, and in some cases attender. If a particular person was contributing part time to the STI clinic work, only that proportion of salary was included in the STI clinic salary cost. For example, if the medical officer was contributing only half time to the STI clinic and the other half to teaching then only half his/her salary was included in the personnel cost. The salary of the personnel was noted from the official records of hospital of which the STI clinic was a part. If fringe benefits were paid in addition to the regular salary, these were included.

The major component of recurrent goods was medicines. The medicines prescribed to patients at these public clinics are provided free. In order to calculate the cost of medicines, first the lowest market retail rates of the medicines usually prescribed at these public clinics for each STI diagnosis during the 2003–2004 were obtained. This was discounted by 30%, which is the estimated discount for bulk purchase of medicines by the government as suggested by the procurement agency. These rates were then applied to the number of each type of STI reported by a STI clinic. The experience of the STI incharge at the Andhra Pradesh State AIDS Control Society suggested that complicated cases needed further treatment and that there were variations in treatment regimens, which would enhanced the overall medication cost by 25%. Accordingly, this enhancement was applied to obtain the total medicine cost for each STI clinic. The other recurrent goods utilised at the STI clinics included inpatient food expenses, test kits for STIs, male condoms, behavioural change communication materials, needles and syringes, gloves, spirit, sodium hypochlorite solution, cotton, soap, dettol, phenyl, distilled water, test tubes, antigen, blotting paper, alcohol, liquid paraffin, buffer solution, stationery and some other laboratory materials and miscellaneous items. The inpatient food expenses were calculated based on cost estimates for items on the menu and the number of inpatients. The test kits for STIs were costed by applying 30% discount to the lowest market rate for bulk purchase by government, as for the medicines. The market price of the condoms supplied to the STI clinic is subsidized by 70% by the government. We considered as the economic cost of the condom what it would have been without the subsidy. Information was obtained from the Andhra Pradesh State AIDS Control Society about the cost of the behavioural change communication materials supplied to the STI clinic, which was used for our analysis. Attempts were made to get the cost of the other goods from the STI clinic records, which if available were used for analysis. If not available, three quotations for these goods were obtained from the market for the 2003–2004 fiscal year and the average of these taken as the cost.

Capital goods used for the work of STI clinics included outpatient and inpatient furniture, electrical fixtures, refrigerator, centrifuge, microscope, needle and syringe destroyer, water bath, slide rotator, water filter, sterilizer, and weighing machine. As for recurrent goods, if information about the cost of capital goods was not available from the STI clinic or its parent hospital, the market price was determined from retail sellers of these goods. The life of the capital goods was assumed to be five years, and therefore, one-fifth of the cost was allocated to the 2003–2004 fiscal year if the good was used for the full year. If a good was purchased in the middle of this fiscal year and used only for half the year, the cost allocated to this good was half of the yearly cost. If a capital good, for example refrigerator or centrifuge, was also being used for work other than that of the STI clinic, a determination was made from the STI clinic staff about the proportion of use for STI work and that proportional cost was allocated to the STI clinic.

Recurrent services included cleaning and building maintenance, electricity, water, telephone, gas/oil, waste disposal, the occasional training of staff during that fiscal year, and some miscellaneous items. The cost of building maintenance was calculated based on the space occupied by the outpatient and inpatient components of the STI clinic. Electricity and water costs were based on applying the market rates to the estimated usage. The estimated usage of electricity was calculated from the electrical fixtures and the number of hours of use per day and for water from the daily estimated use by clinic staff and patients. Telephone and other recurrent services costs were calculated based on actual expenditure.

All STI clinics were located in a parent public hospital, and no rent was being paid. In order to calculate economic rental cost, the actual floor area occupied by the outpatient and inpatient services of the STI clinic was determined, rent rates obtained from three sources in that area for health facilities for the 2003–2004 period, and the average of these rates applied to this area.

For area hospitals, where STI care is generally provided as part of the general outpatient, the costs for STI care were calculated based on estimates of the proportion of personnel time, goods, and services used for STI care, and the rental cost was apportioned based on the ratio of STI cases to all outpatient cases. This estimation did not pose substantial difficulties.

The average exchange rate of Indian Rupees (INR) 45.95 to a US$ for the 2003–2004 fiscal year was used to convert the INR cost to US$ [11].

### Output data

Detailed data were obtained from the written monthly summary records of the STI clinics regarding the services provided every month. These included the number and type of STIs detected and treated, total patient visits, type of tests done, treatment given for STIs, and number of patients receiving inpatient services for STIs and inpatient days. For area hospitals, where STI care is generally provided as part of the general outpatient, data on visits related to STIs were taken from the outpatient records that specify the diagnoses for the visits. Characteristics of programme operation were ascertained, including client characteristics and whether there were any hurdles to the provision of services.

At each STI clinic, 20 patients using the STI services were interviewed regarding their perceptions about these services. The first patients available to the investigators at the STI clinic during the week of data collection were selected for interview, after obtaining verbal informed consent that explained the purpose of the interview and assured anonymity of the respondent. The interviews were conducted in a quiet corner out of the hearing range of others in order to encourage honest responses.

### Quality control

Quality control measures included a thorough pilot study before commencing formal data collection, comprehensive training of a qualified data collection team including their conceptual understanding of all data issues, full back-up and justification for any data recorded, supervision of data collection at each STI clinic by the project coordinator, thorough review by the study team of the data obtained at each STI clinic, and contacting the STI clinics again to obtain information about data issues that needed clarification after the review.

### Data analysis

Analysis of the data was done using SPSS statistical software. The average economic cost per patient diagnosed and treated for STI was calculated as the measure of cost-efficiency of each STI clinic. The relation of this measure of efficiency with the cost components was assessed through regression analyses. Incremental costs for initial and follow-up visits were assessed using a multiple regression model. The personnel, capital goods and rental costs were relatively fixed for each STI clinic irrespective of the volume of services provided. We therefore treated these as relatively fixed costs, and treated the recurrent goods and recurrent services as variable costs for each clinic. In addition to using the number of initial and follow-up visits as independent variables for each STI clinic, we also used the fixed costs as a variable in the right side of the equation as the constant alone may not estimate the entire fixed costs [12]. We used this regression model to assess the total cost function. A high R^2 ^value would be expected for this model, since the left side of the equation (total cost) was determined in our costing largely based on information present in the right side of the equation. That is, fixed costs are clinic-specific and a major portion of variable costs (medicines) was calculated assuming standard inputs and costs per visit across clinics. This is consistent with the objective of our model, which was to determine the incremental costs for initial and follow-up visits.

The optimal number of patients that could have STI detected and treated in a year was calculated using the following assumptions, which were based on input of STI clinic staff and persons familiar with the working of these STI clinics:

1. The number of STIs detected and treated depended mainly on the availability of full-time equivalents of the doctor in the outpatient of the STI clinic. Since the majority of the patients attending these clinics have STIs, the proportion of initial visits not related to STIs is small. Therefore, the latter would not have a substantial impact on the number of STIs that could be detected and treated per full-time equivalent of doctor.

2. An average of two follow-up visits for each STI, i.e. a total of three visits for each STI diagnosis, would be useful for quality care for patients attending these clinics.

3. During the usual 5 working hours of the STI clinic outpatient in a day, a doctor could satisfactorily see 5 patients with new STI diagnosis and 10 follow-ups, without compromising quality.

4. Considering 250 working days in a year, for each doctor full-time equivalent available in the outpatient clinic, 1250 new STIs could be diagnosed and treated without compromising quality.

The economic cost for providing services to the optimal number of STI patients was then estimated.

Reliable data on the number of STIs detected and treated at one of the sampled STI clinics located in an area hospital could not be obtained. Therefore, this clinic was excluded from the analysis and data are presented for 14 of the 15 sampled STI clinics.

## Results

A total of 18807 diagnoses and associated treatment for STIs were reported by the 14 STI clinics in the 2003–2004 fiscal year, with a median value of 1199 and mean of 1343 (Table 1). This number was generally highest at STI clinics located in the tertiary hospitals of medical colleges, followed by the STI clinics in district headquarter hospitals, and least in the STI clinics in area hospitals. This trend was largely due to the differences in the relative sizes of the catchment populations for these three categories. Of the total STIs detected and treated, 59.2% were in males. The male to female ratio was highest at 3:2 for medical college hospital STI clinics but was reverse in area hospital STI clinics (Table 1). The ratio of total out-patient visits to the cases of STIs detected and treated was reported to be highest by the medical college hospital STI clinics (3.33) as compared with the district headquarter hospital STI clinics (1.40) and for the area hospital STI clinics (1.76), suggesting that the number of follow-up visits is relatively higher in the medical college STI clinics. Data regarding the exact reason for each visit were not available. However, it is estimated that the majority of the initial visits in these clinics result in STI diagnoses, and the majority of other visits are follow-up visits related to STI treatment and counselling.

**Table 1:** Number of STIs treated at STI clinics in the fiscal year 2003–2004.

**STI clinic**	**STIs diagnosed and treated**					**Total visits**	**Ratio of total visits to STIs treated**
			
	**Total number**	**Male**		**Female**			
				
		**Number**	**Percent**	**Number**	**Percent**		
**Medical college hospitals**							
MC1	2784	1827	65.6	957	34.4	9093	3.27
MC2	1078	792	73.5	286	26.5	7260	6.73
MC3	1921	1189	61.9	732	38.1	7204	3.75
MC4	2186	1514	69.3	672	30.7	7064	3.23
MC5	1743	1333	76.5	410	23.5	4638	2.66
MC6	2223	1255	56.5	968	43.5	4543	2.04
All MC	11935	7910	66.3	4025	33.7	39802	3.33
**District headquarter hospitals**							
DHQ1	1759	851	48.4	908	51.6	2058	1.17
DHQ2	1320	788	59.7	532	40.3	1365	1.03
DHQ3	1012	447	44.2	565	55.8	1530	1.51
DHQ4	323	257	79.6	66	20.4	1220	3.78
All DHQ	4414	2343	53.1	2071	46.9	6173	1.40
**Area hospitals**							
AH1	551	76	13.8	475	86.2	650	1.18
AH2	582	253	43.5	329	56.5	1215	2.09
AH3	558	220	39.4	338	60.6	631	1.13
AH4	767	331	43.2	436	56.8	1842	2.40
All AH	2458	880	35.8	1578	64.2	4338	1.76
**Total**	18807	11133	59.2	7674	40.8	50313	2.68

All sampled medical college hospitals and most district headquarters hospitals had specialist(s) trained in STIs available for outpatients in their STI clinics, whereas most area hospitals did not have a specialist for their STI clinics. The diagnosis of STIs was most frequently made based on clinical assessment without complete laboratory investigations. Investigations were done most often in STI clinics in medical college hospitals, infrequently in district headquarter hospitals, and almost never in area hospitals. The diagnoses of STIs reported by these STI clinics are shown in Table 2. Of the classic STIs, the diagnosis of herpes was reported to be the highest. Considering all diagnoses together, the highest reported diagnosis was the miscellaneous category that included mostly scabies and some pediculosis and others.

**Table 2:** Distribution of reported STI diagnoses.

**No.**	**Type of STI**	**Number**	**Percent**
1	Herpes	2507	13.3
2	Chlamydial infection	2212	11.8
3	Gonorrhea	1389	7.4
4	Nonspecific genital ulcer	1249	6.6
5	Nonspecific vaginal discharge	1139	6.1
6	Candidiasis	1123	6.0
7	Syphilis	1007	5.4
8	Genital warts	808	4.3
9	Trichomonas	643	3.4
10	Chancroid	586	3.1
11	Lymphogranuloma venereum	228	1.2
12	Bacterial vaginosis	225	1.2
13	Donovanosis	160	0.9
14	Miscellaneous including scabies and pediculosis	5532	29.4
	Total	18807	100

The total economic cost of services provided by the 14 STI clinics during the 2003–2004 fiscal year was INR 13,719,992 (US$ 298,585), of which 9.9% was for inpatient services provided for 1106 (5.9%) of the total 18807 STIs treated. Of the total cost, personnel made up 76.2%, recurrent goods 10.2% (58.5% of this was for STI medicines), rentals 9.9%, recurrent services 2.1%, and capital goods 1.7%. There were modest variations in these proportional costs among some of the STI clinics (Table 3). If the financial costs were considered, by excluding the costs for rentals and condom subsidy, these would be 10.4% less than the economic costs for the 14 STI clinics combined.

**Table 3:** Economic cost of STI clinics in the 2003–2004 fiscal year.

STI clinic*	**Total economic cost**		**Percent of economic cost**					**Percent cost due to inpatient services†**	**Number of STIs treated**	**Cost per STI treated**	
				
	**INR**	**US$**	**Personnel**	**Recurrent goods**	**Rentals**	**Recurrent services**	**Capital goods**			**INR**	**US$**
**Medical college hospitals**											
MC6	1283694	27937	76.5	12.2	6.2	2.7	2.4	8.3	2223	577.5	12.6
MC5	1345786	29288	82.7	7.4	6.7	1.7	1.6	2.6	1743	772.1	16.8
MC4	1746668	38012	75.0	10.0	11.3	1.8	1.9	9.8	2186	799.0	17.4
MC3	1569840	34164	80.6	9.7	6.0	2.0	1.7	5.3	1921	817.2	17.8
MC2	1061341	23098	66.8	8.8	19.2	3.0	2.1	10.2	1078	984.5	21.4
MC1	3345020	72797	79.8	8.0	9.9	1.2	1.1	17.2	2784	1201.5	26.1
All MC	10352349	225296	77.7	9.1	9.6	1.9	1.6	10.4	11935	867.4	18.9
**District headquarter hospitals**											
DHQ1	396627	8632	63.9	25.3	6.4	2.9	1.4	11.0	1759	225.5	4.9
DHQ2	349816	7613	62.5	22.7	8.2	3.4	3.3	15.0	1320	265.0	5.8
DHQ3	612111	13321	73.2	9.8	14.1	1.8	1.1	9.1	1012	604.9	13.2
DHQ4	800543	17422	75.3	4.9	16.7	1.8	1.3	5.2	323	2478.5	53.9
All DHQ	2159097	46988	70.5	12.9	12.7	2.2	1.6	9.0	4414	489.1	10.6
**Area hospitals**											
AH2	198736	4325	60.2	18.4	11.9	5.9	3.6	14.2	582	341.5	7.4
AH4	329889	7179	71.5	14.4	9.0	2.8	2.2	9.7	767	430.1	9.4
AH3	340348	7407	78.7	11.2	5.4	3.0	1.7	6.2	558	609.9	13.3
AH1	339572	7390	76.0	14.2	5.0	3.0	1.7	0.6	551	616.3	13.4
All AH	1208545	26301	72.9	14.1	7.4	3.4	2.2	6.9	2458	491.7	10.7

**Total**	13719992	298585	76.2	10.2	9.9	2.1	1.7	9.9	18807	729.5	15.9

INR is Indian Rupee

1 US$ = INR 45.95 (average exchange rate in the 2003–2004 fiscal year) [11]

*STI clinics arranged within each hospital type category in decreasing order of cost-efficiency, i.e. increasing order of cost per STI treated.

†This inpatient cost does not include cost of medicines for treatment of STIs, which would have been incurred even if these patients were treated as outpatient.

The economic cost for each patient detected to have STI and treated for it varied 5-fold between 13 of the 14 STI clinics from INR 225.5 (US$ 4.91) to INR 1201.5 (US$ 26.15), with a median of INR 609.9 (US$ 13.27) (Table 3). The remaining STI clinic had an unusually high cost per STI detected and treated (INR 2478.5, US$ 53.94). The average cost of detecting and treating each STI for patients at all the 14 STI clinics combined was INR 729.5 (US$ 15.88). The average cost of detecting and treating each STI was 77% higher at the STI clinics in the medical colleges compared with those at district headquarter and area hospitals (Table 3).

**Figure 1:**
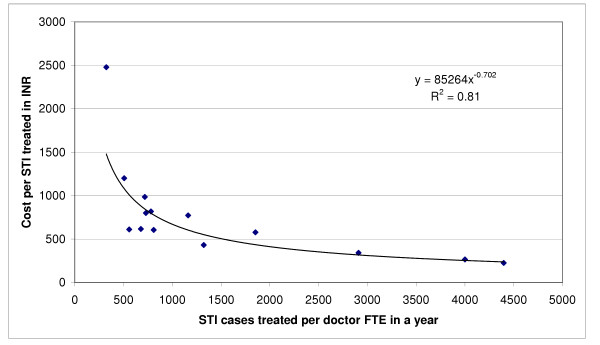
Relationship between STIs treated per doctor full-time equivalent and the cost per STI treated (p < 0.001, power function). FTE is full-time equivalent, INR is Indian Rupee.

**Figure 2:**
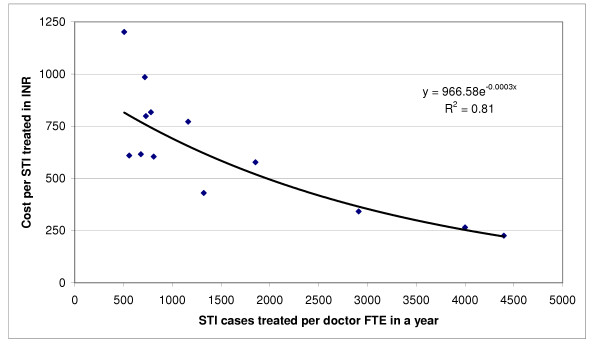
Relationship between STIs treated per doctor full-time equivalent and the cost per STI treated after excluding one extreme value that could be considered an outlier (p < 0.001, exponential function). FTE is full-time equivalent, INR is Indian Rupee.

Since personnel accounted for the major portion of cost, and doctors had the highest relative salary among personnel, there was a significant direct non-linear relation between the number of STI cases treated per doctor full-time equivalent in a year and the cost-efficiency for each STI case treated at the 14 STI clinics, the best fit for which was obtained with a power function (p < 0.001, R^2 ^= 0.81) as shown in Figure 1. If data for the one clinic with an exceptionally high cost per STI treated were excluded (DHQ4), considering it as an outlier, the best fit for this relation was obtained with an exponential function (p < 0.001, R^2 ^= 0.81) as shown in Figure 2.

Considering the 2003–2004 fiscal year data from each STI clinic as a data point, the multiple regression model explained in the methods section was used to assess total economic cost as a function of fixed costs, cost of initial visits in which STI cases were detected (including treatment cost), and cost of other visits (mostly follow-up visits), which revealed the following relation:

 = 3036.35 + 1.04 X + 55.57 Y + 3.75 Z

where  is total economic cost in INR, X is fixed costs, Y is the number of initial visits in which STI cases were detected and treatment/medicines given, and Z is the number of other visits. As expected, this model had a high R^2 ^value of almost 1.00. The fit of the model was significant at p <0.001 (F = 15906; degrees of freedom: 3 for regression, 10 for residual, 13 total). In this model, two variables (fixed costs and initial visits) were statistically significant whereas one variable (follow-up visits) was not (Table 4). This model suggests that apart from the constant/fixed costs, the additional cost for each initial visit in which STI was detected and treatment given (including cost of treatment) was INR 55.57 (US$ 1.21) and the additional cost for each follow-up visit was very small at INR 3.75 (US$ 0.08).

**Table 4:** Coefficients in the multiple regression model and their significance.

**Variable**	**Coefficient (βi)**	**Standard error**	**t**	**Significance**
Constant	3036.35	8049.64	0.377	0.714
Fixed costs	1.04	0.01	95.761	0.000
Visits in which STI was detected	55.57	8.28	6.712	0.000
Other visits (mostly follow-up)	3.75	2.88	1.300	0.223

Dependent variable: Total cost

Personnel at 13 of the 14 (92.9%) STI clinics responded that more patients could be served by their STI clinics with the available personnel and infrastructure if there were more demand. Based on the assumptions mentioned in the methods section about the number of STI cases that could be detected and treated without compromising quality by each doctor in a year if there were optimal demand, 4 of the 6 medical college STI clinics, 2 of the 4 district headquarter STI clinics, and 2 of the 4 area hospital STI clinics could increase the number of STIs detected and treated per year substantially by 54–287% with the available personnel and infrastructure (Table 5). If this were achieved, the overall number of STI cases detected and treated by the 14 STI clinics could increase by 38% from 18807 to 25916. The total cost for this would increase only by 3.5% from the 2003–2004 cost of INR 13,719,992 (US$ 298,585) to INR 14,203,222 (US$ 309,102), using the equation  = 3036.35 + 1.04 X + 55.57 Y + 3.75 Z from the multiple regression model mentioned previously for incremental costs.

**Table 5:** Optimal number of STIs that could be treated at the STI clinics.

STI clinic*	**Doctor full-time equivalents available**			**Number of STIs treated in fiscal year 2003–2004**	**Optimal number of STIs that could be treated†**	**Percent increase if optimal number treated**
				
	**Total**	**Male (%)**	**Female (%)**			
**Medical college hospitals**						
MC6	1.20	1.20 (100)	0 (0)	2223	1500	
MC5	1.50	1.25 (83.3)	0.25 (16.7)	1743	1875	8
MC4	3.00	1.50 (50.0)	1.50 (50.0)	2186	3750	72
MC3	2.46	1.96 (79.7)	0.50 (20.3)	1921	3075	60
MC2	1.50	1.50 (100)	0 (0)	1078	1875	74
MC1	5.50	5.00 (90.9)	0.50 (9.1)	2784	6875	147
All MC	15.16	12.41 (81.9)	2.75 (18.1)	11935	18950	59
**District headquarter hospitals**						
DHQ1	0.40	0.40 (100)	0 (0)	1759	500	
DHQ2	0.33	0 (0)	0.33 (100)	1320	413	
DHQ3	1.25	0.25 (20.0)	1.00 (80.0)	1012	1563	54
DHQ4	1.00	1.00 (100)	0 (0)	323	1250	287
All DHQ	2.98	1.65 (55.4)	1.33 (44.6)	4414	3725	
**Area hospitals**						
AH2	0.20	0.20 (100)	0 (0)	582	250	
AH4	0.58	0.33 (56.9)	0.25 (43.1)	767	725	
AH3	1.00	0.70 (70.0)	0.30 (30.0)	558	1250	124
AH1	0.81	0.31 (38.5)	0.50 (61.5)	551	1016	84
All AH	2.59	1.54 (59.5)	1.05 (40.5)	2458	3241	32
**Total**	20.73	15.60 (75.3)	5.13 (24.7)	18807	25916	38

*STI clinics arranged within each hospital type category in decreasing order of cost-efficiency, i.e. increasing order of cost per STI treated.

†Based on the assumption that one full-time equivalent doctor could satisfactorily treat 1250 STIs in a year, as described in the methods section.

In addition to inadequate demand as the major hurdle to provision of services, two STI clinics mentioned inadequate supplies and two clinics mentioned inadequate staffing as hurdles. The two clinics that mentioned inadequate supplies as hurdle (AH1 and AH3) had the highest cost per STI treated among the area hospitals (Table 3) but this was close to the median cost per STI treated for all clinics considered together. One of the clinics that mentioned inadequate staffing as hurdle (DHQ4) had the highest cost of all per STI treated and the other (AH2) had one of the lowest costs of all per STI treated (Table 3). The small proportion of clinics reporting hurdles other than inadequate demand prevent generalisations about the relation of these hurdles to efficiency.

The proportion of full-time equivalents for female doctors available in the STI clinics of medical colleges was much lower than in the STI clinics of district headquarters and area hospitals (Table 5), which would partly be responsible for the relatively lower proportion of female patients treated in the medical college STI clinics (Table 1).

The patient interviews at the STI clinics revealed 70.4% were very satisfied with the services provided, and the remaining were somewhat satisfied, with no one mentioning that he/she was not satisfied (Table 6).

**Table 6:** Satisfaction of patients with services provided by STI clinics.

**STI clinic**	**Total number of patients interviewed**	**Very satisfied**		**Some what satisfied**		**Not satisfied**	
		
		**No.**	**%**	**No.**	**%**	**No.**	**%**
Medical college hospitals	120	79	65.8	41	34.2	0	0
District headquarter hospitals	80	57	71.2	23	28.8	0	0
Area hospitals	80	61	76.2	19	23.8	0	0
Total	280	197	70.4	83	29.6	0	0

All medical college hospital STI clinics and two district headquarter STI clinics (DHQ2 and DHQ4) were old STI clinics (established since 1960s), and the remaining were newly designated STI clinics in 2002. Because major differences in the services and cost-efficiency were observed between STI clinics located in the three categories of hospitals, and the only category that had both old and new clinics was the district headquarter hospitals, separate analysis for old and new clinics was not done.

## Discussion

In the Andhra Pradesh state of India, analysis of 14 public sector STI clinics revealed that the average cost of diagnosing and treating each STI was INR 729.5 (US$ 15.88), and this cost ranged many-fold between the 14 clinics. Fixed costs of the STI clinics made up the predominant proportion, with personnel costs exceeding three-fourths of the total cost. Although it is unusual to think of personnel as fixed costs, this concept applies here as the personnel were employed at the STI clinics regardless of the amount of services delivered. Based on estimates of optimal workload that could be handled by the available personnel, we estimated that a large proportion of these STI clinics could deal with more STI cases without compromising quality, which would increase the number of STIs treated at the 14 clinics by over one-third the number treated in the 2003–2004 fiscal year at a minimal additional cost of about 3.5% for provision of services. This does not include the additional costs that may be involved in increasing the demand for these services. These are difficult to assess and need to be assessed over a period of time, and may or may not be large. Since the public sector STI clinics predominantly serve poor patients in India at almost no direct cost to the patients, it is important that mechanisms be developed to utilise the unused capacity in these clinics. This would result in more poor patients to be served and more efficient use of the public resources available for STI and HIV control.

Substantial differences were found in the services and cost-efficiency between the STI clinics at the three different type of locations, i.e. medical college, district headquarter and area hospitals. The number of STIs treated per full-time doctor equivalent, as well as the cost-efficiency per STI treated, was least in the medical college hospital STI clinics. However, it is important to note that medical college clinics also had the highest number of follow-up visits per STI, availability of STI specialists, and level of investigations for diagnosis, suggesting that the thoroughness of STI care would be higher at these clinics. In addition, the tertiary medical college clinics are estimated to get more complicated patients and also serve a vital role in the teaching of residents and medical students. Therefore, the relatively higher cost per STI treated in medical college clinics is by itself not a negative thing, if it is associated with provision of higher quality care and care to more complicated patients. Attempts at improving cost-efficiency should not be at the expense of quality of care. We calculated the optimal workload that could be handled by the personnel at the STI clinics taking into account the need to maintain quality. The highest relative unused capacity was in the medical college hospital STI clinics. On the other hand, the low number of follow-up visits at the district headquarter and area hospital STI clinics suggests that emphasis on encouraging follow-ups and quality of care is needed. In addition, the exceptionally high number of STI patients treated at two district headquarter hospital STI clinics (DHQ1 and DHQ2) and the high number at one area hospital STI clinic (AH2) per full-time doctor equivalent available suggests that the need for ensuring quality of care must be emphasised, assuming that quality is impaired by an excessive caseload.

The relatively low proportion of female patients treated at medical college hospital STI clinics can perhaps be attributed to the lower proportion of female doctors available at these clinics as compared with district headquarter and area hospital STI clinics, and also to the fact that gynaecology clinics are available at medical college hospitals that are usually staffed by female doctors and many females go to these clinics for STI-related symptoms. Another factor for the relatively higher proportion of female STI patients seen at the area hospitals is the fact that the STI clinics in these hospitals run as part of the general outpatient and not as a distinctly separate clinic, which encourages more females to utilise these services.

Inpatient services are used by the STI clinics to admit patients of very advanced STIs who are quite poor and have a low chance of coming back for follow-up. Although these inpatient services make up 10% of the total cost for 6% of the total STIs treated, this is generally utilised for the STI patients who are most in need and who are at high risk of not completing the treatment and follow-up in the absence of these services, and therefore, may be justified.

The reported distribution of STI diagnoses should be interpreted cautiously in the background that investigations are done almost never at area hospital clinics and infrequently at district headquarter hospital clinics, and that STI specialists are mostly not available at area hospital clinics. The very high proportion of scabies reported by these public sector STI clinics is generally suggestive of the very low socioeconomic status of the patients utilising the services of these clinics.

An encouraging finding in this study was that of the patients interviewed at the STI clinics over two-third were very satisfied with the clinic services and the remaining somewhat satisfied, with no respondent reporting dissatisfaction. This implies that once the patients, who are mostly poor, reach the STI clinic and use its services, they are generally satisfied with the services. An important factor in this perception is likely to be the fact that free medicines are given for treatment.

Consideration of the above mentioned issues would have to be kept in mind while planning methods to enhance utilisation of the unused capacity in the public sector STI clinics in this Indian state such that the quality of services is also adequate. One major issue that comes in the way of more demand for services of these clinics is the stigma that is attached with attending an STI clinic, which is commonly perceived by the society to be associated with poor personal character or other negative attributes. An option that could be explored to reduce this stigma would be to give a more innocuous name to these clinics, such as, family health clinics, and provide STI services as part of a comprehensive sexual health package. Such an approach would require some restructuring, but may be worth considering as a long-term solution for better utilisation of STI services in the public sector hospitals. Another issue that needs to be addressed to increase the utilisation of services at public sector STI clinics is the development of systematic linkages with non-governmental organisations working with high-risk groups such as sex workers, such that they can easily avail the services of these STI clinics.

The majority of outpatient health care in India, including STI treatment, is provided by the private sector [13]. It is also widely believed that the patients who use the public sector STI clinics are predominantly poor and with relatively advanced STIs that have often been unsuccessfully treated elsewhere. In this background, the cost and efficiency analysis presented in this paper pertains to this most vulnerable group. The high rate of 16–30% HIV in the public sector STI clinic attendees who participated in the sentinel surveillance in Andhra Pradesh over the past few years [4] supports the interpretation that these patients are highly vulnerable. It is possible that the cost-efficiency trends for the private sector STI care in India may be different from those in the public sector. However, it may not be unreasonable to assume that the cost-efficiency estimates of public sector STI care apply to those who are at most risk of acquiring or transmitting HIV, and therefore, are important for assessing and comparing the cost-efficiency and cost-effectiveness of the various HIV prevention strategies.

Estimates of cost and efficiency of STI services in India, and their effectiveness in preventing HIV, have not been previously readily available from India [14]. In fact, the cost estimates of STI treatment from the Mwanza study in Tanzania [15] were used previously by the World Bank to estimate the STI care resources needed in India while preparing the loan agreement for supporting India's AIDS control programme [16]. The cost-efficiency estimates presented in this paper could be used to estimate the resources needed for STI care by the public health care system in Andhra Pradesh, and these data also point to some issues which, if addressed, could lead to enhancement in the provision of these services and also their cost-efficiency. These cost and efficiency estimates of STI treatment could also be used to estimate cost-effectiveness of STI treatment for HIV prevention in India either by using published estimates of effectiveness from other parts of the world or by conducting studies on effectiveness of STI treatment in preventing HIV in India. In addition, these cost-efficiency estimates of STI treatment would be compared with similar estimates from other countries in the PANCEA study that are using similar methodology [10], and also with estimates for other HIV prevention strategies in Andhra Pradesh, such as HIV voluntary counselling and testing [17] and HIV prevention programmes for female sex workers [18] using similar methodology. Such standardised cross-country and country-specific cost-efficiency estimates would be very useful in estimating the resources needed for HIV control in specific countries as well as globally with more confidence than has been possible so far with the available data. This is important in the background of the extensive debate in recent times about the resources needed to control HIV.

## Conclusion

In the Indian state of Andhra Pradesh, there is un-utilised capacity in the public sector STI clinics that provide services predominantly to the poor. Efforts to facilitate utilisation of this capacity would be useful, as this would enable more poor patients with STIs to be served at minimal additional cost, and would also reduce the cost per STI treated leading to more efficient use of public resources. Comprehensive and dynamic analysis of the efficiency of HIV prevention services using standardised methods is necessary to make optimal use of the increasing resources that are becoming available for this purpose in India and other parts of the developing world.

## Competing interests

The author(s) declare that they have no competing interests.

## Authors' contributions

LD led the PANCEA study in India, guided the design, data collection and analysis, and wrote the initial draft of this paper. PS contributed to the design, data collection and analysis. TLNP and EM contributed to the design and advised on data collection, analysis and presentation. MCR, AAK, SGPK, YKR and SM contributed to data collection and analysis. MO contributed to the design and advised on analysis. JGK oversaw the PANCEA design and contributed to the analytic design and presentation. All authors read and approved the final manuscript.

## Presentation at meeting

This paper was presented at the Annual Meeting of the Global Forum for Health Research – Forum 9, Mumbai, India, 12–16 September 2005.

## Pre-publication history

The pre-publication history for this paper can be accessed here:


